# Long-term persistence of withdrawal of temazepam, zopiclone, and zolpidem in older adults: a 3-year follow-up study

**DOI:** 10.1186/s12877-018-0829-9

**Published:** 2018-06-15

**Authors:** Juha Puustinen, Ritva Lähteenmäki, Janne Nurminen, Tero Vahlberg, Pertti Aarnio, Markku Partinen, Ismo Räihä, Pertti J. Neuvonen, Sirkka-Liisa Kivelä

**Affiliations:** 1 Satakunta Hospital District, Satakunta Central Hospital, Unit of Neurology, Sairaalantie 3, 28500 Pori, Finland; 2 Division of Pharmacology and Pharmacotherapy, Clinical Pharmacy Group, University of Helsinki, Helsinki, Finland; 3 Unit of Family Medicine, University of Turku, Turku, Finland; 4 The Medical Imaging Centre of Southwest Finland, Turku University Hospital, Turku, Finland; 5 Department of Biostatistics, University of Turku, Turku, Finland; 6 Satakunta Hospital District, Satakunta Central Hospital, Unit of Surgery, Pori, Finland; 7 Helsinki Sleep Clinic, Vitalmed Research Center, Helsinki, Finland; 8 Department of Clinical Neurosciences, University of Helsinki, Helsinki, Finland; 9 Department of Clinical Pharmacology, University of Helsinki, Helsinki, Finland

**Keywords:** Benzodiazepine agonists, Long-term hypnotic use, Withdrawal persistence, Follow-up study, Older outpatients

## Abstract

**Background:**

Studies on persistence of benzodiazepine agonist (BZDA) withdrawal in older outpatients are few, and few studies on long-term persistence over years have yet been published. To describe the persistence of temazepam, zolpidem, and zopiclone (BZDA) withdrawal among older outpatients at 3 years from the beginning of withdrawal, as well as any changes in use of other medications.

**Methods:**

92 outpatients (≥55 years) with primary insomnia, long-term BZDA use as hypnotics (mean duration of BZDA use 9.9 ± 6.2 years), and willingness to withdraw from BZDAs each received either melatonin or a placebo nightly for one month. During this period, BZDAs were meant to be gradually withdrawn. Sleep hygiene counselling and psychosocial support were provided. Three years later, use of BZDAs and other medications was determined by interview and confirmed from medical records.

**Results:**

Of the original 92 outpatients, 83 (90%) participated in the 3-year survey (mean follow-up 3.3 ± 0.2 years). The number of BZDA-free participants decreased from 34 (37%) at 6 months to 26 (28%; intention-to-treat) at 3 years, that of irregular BZDA users decreased from 44 (48%) at 6 months to 27 (29%) at 3 years, while that of regular users increased from 11 (12%) at 6 months to 30 (33%) at 3 years (*P* = 0.001).

Those who were regular BZDA users at 3 years had at baseline (before withdrawal) higher BMI (*P* = 0.001) than did other participants. At 3 years, the total number of medications remained unchanged for non-users (*P* = 0.432), but increased for the irregular (*P* = 0.011) and regular users (*P* = 0.026) compared to baseline. At 3 years, compared to baseline, use of antidepressants, dopamine agonists, melatonin, and NSAIDs/paracetamol was significantly more common in the whole cohort, but their use did not differ between the BZDA-user subgroups. Randomization to melatonin or placebo during BZDA withdrawal was unrelated to BZDA-withdrawal result.

**Conclusions:**

At 3 years after withdrawal, the number of BZDA-free participants had decreased, but still one-third of the subjects remained BZDA-free, and one-third had reduced their use. Successful BZDA withdrawal did not lead to any increase in total number of medications; use of symptomatic medications in the whole cohort, however, did increase.

## Background

Long-term benzodiazepine agonist (BZDA) use can result in adverse outcomes such as increased risk of falls, fractures, cognitive decline, and mortality [1–3]. Prolonged benzodiazepine use as a hypnotic is considered inappropriate, according to guidelines on pharmacotherapy in the aged [4]. However, a considerable proportion of subjects with insomnia using BZDA hypnotics on a nightly basis for years do still experience sleep disturbances despite hypnotic medication [5, 6]. In a primary-care population, prevalence of BZDA hypnotic drug prescriptions is much higher for multimorbid patients than for patients without multimorbidity [7]. The prevalence of chronic BZDA use is particularly high in nursing-home residents, it has ranged in European nursing homes from 28% [8] to more than 50% [9].

Meta-analyses of interventions for reducing inappropriate long-term BZDA use in older adults have shown supervised BZDA withdrawal augmented with psychotherapy to be the most effective intervention, but for pragmatic reasons, a patient-centred approach with individual planning and monitoring or medication reviews is the recommendation [10, 11]. Very little data on the long-term persistence of BZDA withdrawal results exist, as most of the follow-ups have been short, 0.5 to 3 months [11]. Few studies have reported follow-ups lasting 12 months or longer [11–13]. Morin et al. did a 24-month outcome study among community-dwelling residents who had used as hypnotics various benzodiazepine medicaments but not the widely used Z-drugs: zopiclone or zolpidem [14]. Vicens et al. [15] compared, in a 3-year study, educational methods with routine care in regard to cessation of benzodiazepine use. However, most of their patients seem to have used benzodiazepines for indications other than as hypnotics, and a medical withdrawal intervention group was lacking. Furthermore, whether BZDA withdrawal can affect the use of other central nervous system (CNS) -affecting drugs remains unknown. Our outpatient study is the first in which persistence of BZDA hypnotic (mainly Z-drugs) discontinuation has been followed for up to 3 years in chronic BZDA users, after melatonin/placebo-supported initial withdrawal.

We performed in older outpatients a psychosocially supported BZDA withdrawal intervention as described earlier [16]. BZDA withdrawal rapidly improved muscle strength and balance, but failed to improve cognitive performance [17, 18]. The present secondary data analysis aimed to study in patients of our original cohort their long-term persistence of BZDA withdrawal and possible changes in their medications. In short, our aim was to describe the persistence of temazepam, zolpidem, and zopiclone (BZDA) withdrawal among older outpatients at 3 years from the beginning of withdrawal, as well as any changes in their use of other medications.

## Methods

Details of this temazepam, zopiclone, and zolpidem withdrawal study, its participants, interventions, measurements, and withdrawal results up to 6 months have appeared elsewhere [16–18]. In short, the original Satauni study was a randomized, double-blind, placebo-controlled, parallel-group study on the efficacy of daily melatonin (2 mg) in BZDA withdrawal during a one-month period and during a double-blind 5-month follow-up. At baseline, a physician provided individual psychological support and sleep-hygiene counselling, including discussions about regular sleep rhythm and factors influencing sleep. The psychological support for all participants was continued by a nurse who provided a supportive visit once a week and was available by phone during the one-month withdrawal (period). The participants had follow-up meetings with a nurse at months 2 and 6, and with a physician at month 6 after withdrawal initiation. After this, the participants returned to normal, routine care at their health centres.

The main inclusion criteria at baseline were primary insomnia according to the criteria of the Diagnostic and Statistical Manual of Mental Disorders, 4th edition (DSM-IV) [19], age ≥ 55 years, and long-term (> 1 month) regular night-time use of temazepam, zopiclone, or zolpidem to treat primary insomnia. The key exclusion criteria were BZDA use other than those uses identified above, current use of antipsychotic or antiepileptic medication, active alcohol or drug abuse or a history of abuse, anxiety disorder or other psychiatric disorder, neurological disease, smoking more than ten cigarettes a day, or autoimmune disease [16].

For the present 3-year follow-up study, we sent a letter to all our 92 initial participants and invited them for a follow-up meeting and measurements. The study nurse (JS, MS) met the participants and interviewed them. If participants were unable to schedule an in-person meeting, the study nurse called each one on the telephone and requested return of the completed questionnaire.

The Satauni study protocol was approved by the Ethics Committee of Satakunta Hospital District (2§/7/2008) and by the National Agency for Medicines of Finland (218/2008) and registered to EudraCT (2008–0006795-30). Written informed consent was received from each participant before the study began.

### Measurements and data collection

The participants were asked to report, as part of the questionnaire, all medicines used. The study nurse verified the data by interviewing and examining the medical records. Full medication lists were collected at baseline and at the 3-year follow-up point, BZDAs were collected at baseline, at one and 6 months, and at the 3-year follow-up points. Structured questionnaires provided demographic data (age, gender, body mass index), use of alcohol, duration of BZDA use, exercise activity, living conditions, educational level, occupational status, driver’s licence, smoking, satisfaction with life, self-reported health, and expected health 1 year later. Depressive symptoms were measured with the Geriatric Depression Scale 15-point version (GDS-15) [20].

### Statistical analyses

For our persistence analysis of BZDA withdrawal results, participants were categorized to non-users (no BZDA use), irregular users (intermittent BZDA use but no daily use), and regular users (daily users of BZDA) according to their BZDA use at the 3-year follow-up. The percentages of non-users, irregular users, and regular users were calculated according to the intention-to-treat principle from the number of original participants, 92.

For statistical analysis, each drug was coded according to the Anatomical Therapeutic Chemical Classification of Medicines (ATC) [21] codes and grouped into one of the following groups: BZDA (N05BA, N05CD, N03AE01, N05CF, A03CA), antipsychotics (N05A), antidepressants (N06A, N06CA), antiepileptics and gabapentinoids (N03A), dopamine agonists (N04 BC), melatonin (N05CH01), opioids (N02A, R05DA, R05FA), antihistamines (R06A, N05BB), and anticholinergics (see ref. [22] for full list). The “CNS medication” variable combined these, excluding BZDA and melatonin and a combination variable for non-steroidal anti-inflammatory drugs (NSAID) (M01A) and paracetamol (N02BE01).

Differences in baseline measurements between non-users, irregular users, and regular users were tested by chi-square and Fisher exact tests for variables measured with nominal or ordinal scales, and by the Kruskal-Wallis test for those non-normally distributed, or by one-way analysis of variance with Tukey’s post-hoc tests for normally distributed continuous variables. The normality of the distributions was tested by the Shapiro-Wilk test. The Kruskal-Wallis test served to test differences in the number of all medications between groups; changes within groups were tested with the Wilcoxon signed rank test. Changes in medication use were analyzed with McNemar’s test, and logistic regression analysis using generalized estimating equations.

*P*-values less than 0.05 were considered statistically significant. The statistical analyses were performed with SAS version 9.4 (SAS Institute Inc., Cary, NC, USA).

## Results

### Patient characteristics

**Fig. 1:**
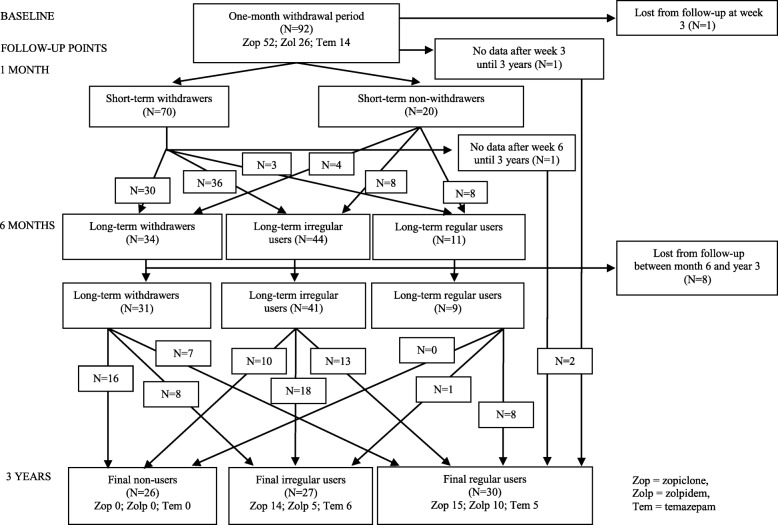
Flow of participants through the study: From recruitment to completion

Of those 92 who originally enrolled in the study, 83 (90%) participated in the 3-year follow-up interview. At baseline, mean BZDA use was 9.9 ± 6.2 years. Nine participants (10%) were lost during follow-up (Fig. 1). Of these, two participants died, and one moved away before the follow-up meeting. The mean follow-up time after the beginning of BZDA withdrawal was 3.3 ± 0.2 years.

Baseline characteristics of the participants, grouped according to use of BZDA at the 3-year follow-up, are in Table 1. Regular BZDA users at 3 years had significantly higher BMI (body mass index) at baseline than did non-users (*P* = 0.004) or irregular users (*P* = 0.005). Additionally, expected health a year later was less uniform in non-users and irregular users than in regular users (*P* = 0.001). The participants’ characteristics, including the BZDA (temazepam, zopiclone, zolpidem) they had used as a hypnotic, were, however, poor predictors of long-term withdrawal persistence (Fig. 1, Table 2).

**Table 1:** Characteristics of participants at baseline and grouped by BZDA use data at the 3-year follow-up point (final non-users, irregular users, and users of BZDA)

	Non-users (*N* = 26)		Irregular users (*N* = 27)		Regular users (*N* = 30)		P
	Mean ± SD		Mean ± SD		Mean ± SD		
Age (years)	66.3 ± 5.6		66.9 ± 6.2		65.9 ± 6.9		0.815
Body Mass Index (kg/m^2^)*	26.4 ± 3.8		26.5 ± 2.9		30.0 ± 4.9		0.001
	Median [LQ, UQ]		Median [LQ, UQ]		Median [LQ, UQ]		
Use of alcohol (doses/week)	0.9 [0, 5.5]		2.5 [0, 5.3]		0 [0, 2.0]		0.106
	N	(%)	N	(%)	N	(%)	
BZDA use at baseline							
< 5 years	3	(12)	3	(11)	4	(13)	0.989
5–10 years	13	(50)	13	(48)	16	(53)	
≥ 10 years	10	(39)	11	(41)	10	(34)	
Randomized to CRM/placebo during withdrawal	12/14	(46/54)	13/14	(48/52)	17/13	(57/43)	0.700
Gender, women/men	16/10	(62/38)	16/11	(59/41)	25/5	(83/17)	0.094
Exercise activity							
Low	1	(4)	2	(7)	3	(10)	0.696
Medium	25	(96)	23	(85)	26	(87)	
High	0	(0)	2	(7)	1	(3)	
Living conditions							
With another person	17	(65)	23	(85)	21	(70)	0.228
Alone	9	(35)	4	(15)	9	(30)	
Education level							
Basic	12	(46)	9	(33)	17	(59)	0.252
Middle grade	10	(38)	16	(59)	9	(31)	
College or academic	4	(15)	2	(7)	3	(10)	
Occupational status							
Regular day shifts	4	(15)	1	(4)	6	(20)	0.357
Irregular day shifts, shift-work	2	(8)	1	(4)	2	(7)	
Retired or unemployed	20	(77)	25	(93)	22	(73)	
Drivers’s licence	23	(88)	24	(89)	26	(87)	1.000
Depression							
Not depressed (GDS-15 sum score < 6)	25	(96)	25	(93)	24	(80)	0.139
Depressed (GDS-15 sum score ≥ 6)	1	(4)	2	(7)	6	(20)	
Smoking							
Non-smoker	25	(96)	27	(100)	26	(87)	0.098
Smoker	1	(4)	0	(0)	4	(13)	
Use of alcohol							
Non-user	4	(15)	5	(19)	7	(24)	0.192
Once a month or more seldom	9	(34)	5	(19)	14	(48)	
2–4 times a month	7	(27)	11	(42)	6	(21)	
2 times a week or more often	6	(23)	5	(19)	2	(7)	
Satisfaction with life							
Very satisfied	4	(15)	4	(15)	2	(7)	0.871
Quite satisfied	14	(54)	16	(59)	16	(53)	
Not satisfied, but not unhappy	6	(23)	6	(22)	10	(33)	
Quite unhappy	2	(8)	1	(4)	2	(7)	
Self-reported health							
Good	6	(23)	5	(19)	5	(17)	0.855
Fair	15	(58)	19	(70)	21	(70)	
Poor	5	(19)	3	(11)	4	(13)	
Expected health a year later#							
Healthier than now	6	(23)	3	(11)	13	(43)	0.001
No change	18	(69)	23	(85)	10	(33)	
A bit worse than now	2	(8)	1	(4)	7	(23)	

*Significant differences in final non-users vs. regular users (P = 0.004) and final irregular users vs. regular users (*P* = 0.005)

#Significant differences in final non-users vs. regular users (*P* = 0.029) and final irregular users vs. regular users (*P* < 0.001)

P = Statistical significance of difference between final non-users, final irregular users, and final regular users

*LQ* Lower quartile, *UQ* Upper quartile, *BZDA* Benzodiazepine agonist, *CRM* Controlled-release melatonin, *GDS* Geriatric Depression Scale

**Table 2:** Number of BZDA prescriptions at baseline and at 1-month, 6-month, and 3-year follow-ups grouped by the BZDA used at baseline and withdrawal status data at 3-year follow-up (non-users, irregular users, and regular BZDA users)

			Zopiclone				Zolpidem				Temazepam				Other BZDA				Total BZDA users	
	Non-users of any BZDA		Irregular		Regular		Irregular		Regular		Irregular		Regular		Irregular		Regular			
	N	(%)	N	(%)	N	(%)	N	(%)	N	(%)	N	(%)	N	(%)	N	(%)	N	(%)	N	(%)
Baseline (92)	0	(0)	0	(0)	52	(57)	0	(0)	26	(28)	0	(0)	14	(15)	0	(0)	0	(0)	92	(100)
1-month (90)	70	(78)	8	(9)	1	(1)	4	(4)	0	(0)	6	(7)	1	(1)	0	(0)	0	(0)	20	(22)
6-month (89)	34	(38)	25	(48)	6	(12)	12	(46)	2	(8)	7	(50)	3	(21)	0	(0)	0	(0)	55	(62)
3-year (83)	26	(31)	14	(27)	15	(29)	5	(19)	10	(38)	6	(43)	5	(36)	7^a^	(8)	1^b^	(1)	57	(69)

^a^oxazepam (*N* = 4), alprazolam (*N* = 1), diazepam (*N* = 1) and clonazepam (*N* = 1)

^b^chlordiazepoxide with amitriptyline combination preparation (*N* = 1)

### Persistence of withdrawal results up to 3 years

The number of BZDA-free participants had decreased from 34 (37%) at the 6-month follow-up to 26 (28%) at 3 years, and that of irregular BZDA users from 44 (48%) at 6 months to 27 (29%). The number of nightly regular users had increased from 11 (12%) at months to 30 (33%) at 3 years (*P* = 0.001). Use of melatonin (vs. placebo) during the BZDA withdrawal month was not related to BZDA use after 3 years (Table 1). The persistence of withdrawal from individual BZDAs is in Table 2.

### Change in use of other drugs

BZDA withdrawal did not affect the total number of medications in the non-users’ group (*P* = 0.432) (Table 3). The total number of medications of irregular users (*P* = 0.011) and of regular users increased (*P* = 0.026) compared to baseline. Of the various medications, use of paracetamol or NSAIDs doubled in the whole cohort (*P* < 0.001), but with no significant differences between the final BZDA non-users, irregular users, and regular users. Similarly, use of antidepressants and dopamine agonists increased within 3 years in the cohort, but without a significant difference between these groups. The use of melatonin increased, particularly for the BZDA non-users.

**Table 3:** Use of medications, especially those with CNS effects, at baseline (before withdrawal) and at 3-year follow-up. Participants are grouped by BZDA withdrawal status at 3 years (non-users, irregular users, regular users). P is for baseline vs. 3-year follow-up

	Non-users (*N* = 26)		Irregular users (*N* = 27)		Regular users (*N* = 30)		P (between groups)
	Median	[LQ,UQ]	Median	[LQ,UQ]	Median	[LQ,UQ]	
Number of all medications							
Baseline	4	[3, 5]	4	[4, 5]	4	[3, 6]	0.767
3-year follow-up	4	[2, 6]	5	[3, 7]	5	[3, 7]	0.222
P	0.432		0.011		0.026		
Number of concomitant CNS medications^a^							
Baseline	0	[0, 1]	0	[0, 1]	0	[0, 1]	0.744
3-year follow-up	1	[0, 1]	0	[0, 1]	0	[0, 1]	0.286
P	0.268		0.307		0.827		
Users of medications groups	N	(%)	N	(%)	N	(%)	
Antipsychotics							
Baseline	0	(0)	0	(0)	0	(0)	1.000
3-year follow-up	0	(0)	1	(4)	1	(3)	1.000
P	0.157						
Antidepressants							
Baseline	4	(15)	7	(26)	6	(20)	0.634
3-year follow-up	9	(34)	11	(41)	9	(30)	0.677
P	0.006						
Antiepileptics, gabapentinoids							
Baseline	1	(4)	1	(4)	1	(3)	1.000
3-year follow-up	0	(0)	1	(4)	0	(0)	0.639
P	0.337						
Dopamine agonists							
Baseline	0	(0)	0	(0)	0	(0)	1.000
3-year follow-up	2	(8)	2	(7)	0	(0)	0.382
P	0.046						
Melatonin							
Baseline	0	(0)	0	(0)	0	(0)	1.000
3-year follow-up	7	(27)	3	(11)	4	(13)	0.290
P	0.001						
Opioids							
Baseline	2	(8)	3	(11)	1	(3)	0.507
3-year follow-up	1	(4)	0	(0)	2	(7)	0.643
P	0.190						
NSAIDS, paracetamol^b^							
Baseline	8	(31)	4	(15)	7	(23)	0.384
3-year follow-up	16	(62)	12	(44)	15	(50)	0.447
P	< 0.001						
Antihistamines							
Baseline	4	(15)	1	(4)	2	(7)	0.330
3-year follow-up	4	(15)	1	(4)	2	(7)	0.330
P	1.000						
Anticholinergics							
Baseline	5	(19)	4	(15)	4	(13)	0.867
3-year follow-up	8	(31)	6	(22)	4	(13)	0.287
P	0.252						

^a^number of CNS medications per participant (excl. BZDA and melatonin): antipsychotics, antidepressants, antiepileptics, dopamine agonists, antihistamines, anticholinergics

^b^only aspirin at analgesic doses (500 mg/d or higher) included; aspirin at antithrombotic doses (499 mg/d or lower) excluded

## Discussion

To our knowledge, this is the lengthiest study to ever report BZDA withdrawal results after medical withdrawal intervention (melatonin or placebo) with its follow-up of 3 years. Our cohort had a high follow-up retention rate after a structured BZDA withdrawal intervention in older outpatients, individuals who had for a long time regularly used short-acting BZDAs as hypnotics. Most (85%) of our participants had used chronically short-acting “Z-drugs,” zopiclone or zolpidem, and only 15% had temazepam as their nightly hypnotic. The short-term (at 1 month) withdrawal results in this cohort were good [16]. However, BZDA use increased markedly over time after the withdrawal period.

Three years after the one-month BZDA withdrawal period, roughly one-third of the initial participants remained non-users, one-third used BZDAs irregularly, and one-third took BZDAs regularly as a hypnotic. The withdrawal rates and persistence of the withdrawal seemed to be similar irrespective of the hypnotic. Controlled-release melatonin during the one-month BZDA withdrawal period did not affect the long-term BZDA-withdrawal result. One explanation for persistence of BZDA withdrawal was the psychosocial support during the first month and at 2 and 6 months for all participants. The number of all medications (median four drugs per patient at baseline) did not increase within 3 years in non-users, whereas during that period—for irregular users and regular users—it increased. However, the number of participants using antidepressants, dopamine agonists, melatonin, and NSAIDs/paracetamol increased in all groups. Worryingly, some long-acting benzodiazepines and BZDA combination preparations with anticholinergic antidepressants were prescribed by the time of the 3-year follow-up.

Most patient characteristics at baseline were fairly similar in all groups despite the patients’ differing BZDA use at the 3-year follow-up point. Yet, higher BMI at baseline, i.e., before start of withdrawal, was associated at the 3-year follow-up with regular BZDA use. Higher BMI may be associated with sleep apnoea, but treatment more appropriate than BZDA would be weight loss and positive airway pressure therapy. As no clear markers as yet exist, it is, however, impossible to predict at baseline who will be a successful BZDA withdrawer and who will not.

Expected health between the groups at baseline was not uniform. As there were no major differences in majority background variables, we decided that BMI and expected health were not clinically meaningful background variables to be adjusted for. Furthermore, small sample size would have complicated the multivariate analyses. Of note, most—meaning 23 of the 26 final non-users (at 3 years)—had used BZDA hypnotics regularly for at least 5 years before entering this withdrawal study. Thus, even after their very long-time use, BZDA hypnotics, at the usual therapeutic doses, may not reduce the likelihood of withdrawal of a patient who is well motivated. On the other hand, a clear distinction arises, as pointed out, in withdrawal success between abusers of high doses of BZDAs and those using therapeutic doses as hypnotics [23].

All our patients were outpatients, and nine of them could not be contacted for the 3-year follow-up; at least two of them had died, and one moved away before the 3-year follow-up point. Here, percentages of withdrawal persistence were calculated conservatively by the intention-to-treat (ITT) principle, meaning comparison of numbers withdrawing to those 92 who had entered the withdrawal study. Thus, the true withdrawal persistence could be somewhat better than reported here.

According to recent withdrawal studies, spontaneous BZDA withdrawal rate without any interventions ranges from 5 to 26% [10, 11, 15, 24]. The study other than ours with a 3-year follow-up reporting the results of educational withdrawal intervention [15], with one-time counselling or with counselling combined with follow-up meetings lasting between 2 and 3 weeks, produced respective withdrawal rates of 41 and 39%. In their control group (routine care), 26% had withdrawn [15]. In our study, the BZDA withdrawal rate was 28% at 3 years after psychosocially supported BZDA withdrawal augmented with melatonin.

Persistence of BZDA withdrawal over years might have been better had psychosocial support continued after the one-month period. Additionally, there exist several BZDA withdrawal interventions with shorter follow-ups: In Canada, counselling at a pharmacy produced a 27% persistence rate of BZDA withdrawal and dose reductions for 11% of its participants at 6 months [24]. An American study [25] randomized long-term BZDA users into three groups, among which one group received cognitive behavioural therapy, one placebo therapy (biofeedback), and one received physician-given counselling for gradual BZDA withdrawal. In all groups, the BZDA use decreased 84% compared to baseline, and BZD withdrawal persistence remained at one-third compared to baseline in all groups for up to 1 year [25]. However, no direct comparisons between previous studies can be made due to different withdrawal methods and follow-ups times.

Why does BZDA withdrawal induce increased use of other CNS medications? First, hypnotic drugs have a strong placebo effect [26]. Second, it can be hypothesized that BZDA withdrawal reveals previously untreated conditions. The increase in symptomatic medications can be explained by such symptoms that had been eased by BZDA. Insomnia may precede comorbid depression. The undiagnosed depressive symptoms, depression, and muscle aches or progressive osteoarthritis, as well as sleep problems explain at least partially participants’ increased antidepressant use. By 3 years, melatonin seems to have replaced BZDAs as the hypnotic for some (27%) of the BZDA non-users. Interestingly, melatonin itself, in a recent meta-analysis, did not improve BZDA withdrawal [27]. The minor increase in low-dose pramipexole (dopamine agonist) use in BZDA non-users and in irregular users may be related to its use for restless legs which had contributed to sleep disturbances; in fact, BZDAs may have masked previously unrecognized symptoms of restless legs syndrome.

The main strengths of this study are its long follow-up time of up to 3 years and the very high follow-up retention rate of participants (90%), of whom most had for a lengthy period used zopiclone or zolpidem up until the withdrawal. One potential weakness is the relative small sample size that limits validity of conclusions and does not allow comparisons between individual hypnotics. Another potential weakness is that use of BZDAs and other medications at the 6-month and 3-year follow-up points was based on interview data verified from medical records but not by blood- or urine drug determinations. However, no significant discrepancy between patient-reported BZDA use and plasma levels emerged at baseline or at the one-month follow-up point in these participants [15]. Additionally, we have no data on the medications between the 6-month and 3-year follow-up points.

## Conclusions

Psychosocially supported gradual BZDA withdrawal was effective in discontinuation of long-term hypnotic use, but withdrawal persistence decreased over time. At 3 years after withdrawal, nearly one-third of the previous chronic users were BZDA-free, one-third used it irregularly, and one-third continued nightly use. High BMI seems to predict poor withdrawal persistence, but melatonin given during the withdrawal month failed to improve persistence results.

## Abbreviations

ATC: Anatomical Therapeutic Chemical Classification of Medicines
BMI: Body mass index
BZDA: Benzodiazepine agonist (temazepam, zolpidem, zopiclone)
CNS: Central nervous system
DSM-IV: Diagnostic and Statistical Manual of Mental Disorders, 4^th^ edition
EudraCT: European Union drug regulating authorities clinical trials
GDS-15: Geriatric depression Scale 15-point version
ITT: Intention-to-treat
NSAID: Non-steroidal anti-inflammatory drugs
P: *P*-value
